# Prioritization of genes driving congenital phenotypes of patients with de novo genomic structural variants

**DOI:** 10.1186/s13073-019-0692-0

**Published:** 2019-12-04

**Authors:** Sjors Middelkamp, Judith M. Vlaar, Jacques Giltay, Jerome Korzelius, Nicolle Besselink, Sander Boymans, Roel Janssen, Lisanne de la Fonteijne, Ellen van Binsbergen, Markus J. van Roosmalen, Ron Hochstenbach, Daniela Giachino, Michael E. Talkowski, Wigard P. Kloosterman, Edwin Cuppen

**Affiliations:** 1 Center for Molecular Medicine and Oncode Institute, University Medical Center Utrecht, 3584 CX Utrecht, the Netherlands; 2 Department of Genetics, University Medical Center Utrecht, 3584 EA Utrecht, the Netherlands; 3 Max Planck Institute for Biology of Aging, Cologne, Germany; 4 Medical Genetics Unit, Department of Clinical and Biological Sciences, University of Torino, 10043 Orbassano, Italy; 5 Center for Genomic Medicine, Massachusetts General Hospital, Boston, MA USA; 6 Department of Neurology, Massachusetts General Hospital and Harvard Medical School, Boston, MA USA; 7 Program in Medical and Population Genetics and Stanley Center for Psychiatric Research, Broad Institute of MIT and Harvard, Cambridge, MA USA

**Keywords:** Structural variation, Copy number variants, Neurodevelopmental disorders, Intellectual disability, Multiple congenital anomalies, Driver genes, Whole-genome sequencing, Transcriptome sequencing, Topologically associating domains, Position effects

## Abstract

**Background:**

Genomic structural variants (SVs) can affect many genes and regulatory elements. Therefore, the molecular mechanisms driving the phenotypes of patients carrying de novo SVs are frequently unknown.

**Methods:**

We applied a combination of systematic experimental and bioinformatic methods to improve the molecular diagnosis of 39 patients with multiple congenital abnormalities and/or intellectual disability harboring apparent de novo SVs, most with an inconclusive diagnosis after regular genetic testing.

**Results:**

In 7 of these cases (18%), whole-genome sequencing analysis revealed disease-relevant complexities of the SVs missed in routine microarray-based analyses. We developed a computational tool to predict the effects on genes directly affected by SVs and on genes indirectly affected likely due to the changes in chromatin organization and impact on regulatory mechanisms. By combining these functional predictions with extensive phenotype information, candidate driver genes were identified in 16/39 (41%) patients. In 8 cases, evidence was found for the involvement of multiple candidate drivers contributing to different parts of the phenotypes. Subsequently, we applied this computational method to two cohorts containing a total of 379 patients with previously detected and classified de novo SVs and identified candidate driver genes in 189 cases (50%), including 40 cases whose SVs were previously not classified as pathogenic. Pathogenic position effects were predicted in 28% of all studied cases with balanced SVs and in 11% of the cases with copy number variants.

**Conclusions:**

These results demonstrate an integrated computational and experimental approach to predict driver genes based on analyses of WGS data with phenotype association and chromatin organization datasets. These analyses nominate new pathogenic loci and have strong potential to improve the molecular diagnosis of patients with de novo SVs.

## Background

De novo constitutional structural variations (SVs) including deletions, duplications, inversions, insertions, and translocations are important causes of (neuro-)developmental disorders such as intellectual disability and autism spectrum disorder (ASD) [1, 2]. Clinical genetic centers routinely use microarrays, as well as karyotyping in some cases, to detect SVs at kilo- to megabase resolution [3]. The interpretation of the pathogenicity of an SV mainly relies on finding overlap with SVs in other patients with similar phenotypes [4, 5]. SVs can affect large genomic regions which can contain many genes and non-coding regulatory elements [1]. This makes it challenging to determine which and how specific affected gene(s) and regulatory elements contributed to the phenotype of a patient. Therefore, the causative genes driving the phenotype are frequently unknown for patients with de novo SVs which can hamper conclusive genetic diagnosis.

SVs can have a direct effect on the expression and functioning of genes by altering their copy number or by truncating their coding sequences [1]. In addition, SVs can indirectly influence the expression of adjacent genes by disrupting the interactions with their regulatory elements [6]. New developments in chromatin conformation capture (3C)-based technologies such as Hi-C have provided the means to study these indirect, position effects [7]. Most of the genomic interactions (loops) between genes and enhancers occur within megabase-sized topologically associating domains (TADs). These domains are separated from each other by boundary elements characterized by CTCF-binding, which limit the interactions between genes and enhancers that are not located within the same TAD [8, 9]. For several loci, such as the *EPHA4* [10], *SOX9* [11], *IHH* [12], and *Pitx* [13] loci, it has been demonstrated that disruption of TAD boundaries by SVs can cause rewiring of genomic interactions between genes and enhancers, which can lead to altered gene expression during embryonic development and ultimately in disease phenotypes [14]. Although the organization of TADs appears to be stable across cell types, sub-TAD genomic interactions between genes and regulatory elements have been shown to be relatively dynamic and cell type-specific [15]. Disruptions of genomic interactions are therefore optimally studied in disease-relevant cell types, which may be obtained from mouse models or from patient-derived induced pluripotent stem cells. However, it is not feasible to study each individual locus or patient with such elaborate approaches, and disease-relevant tissues derived from patients are usually not available. Therefore, it is not yet precisely known how frequently position effects contribute to the phenotypes of patients with developmental disorders.

A few computational tools such as SVScore and the Ensembl Variant Effect Predictor have been developed to predict the pathogenicity of SVs, but these mainly predict the potential direct impact of SVs on genes and do not take the specific phenotype of the patient into account [16, 17]. It has been shown that the use of computational methods based on combining phenotypic information from the Human Phenotype Ontology (HPO) database (phenomatching) with previously published chromatin interaction datasets can improve the interpretation of the molecular consequences of de novo SVs [18–20]. These approaches have largely been based on data derived from a small set of cell types and techniques. Here, we further expand these in silico approaches by integrating detailed phenotype information with genome-wide chromatin conformation datasets of many different cell types. By combining this method with whole-genome and transcriptome sequencing, we predicted which genes are affected by the SVs and which of these genes have likely been involved in the development of the disease phenotype (e.g., candidate driver genes). Accurate characterization of the effects of SVs on genes can be beneficial for the prediction of potential clinical relevance of the SVs. Detailed interpretation of the molecular effects of the SVs helped to identify candidate driver genes in 16 out of 39 patients who had an inconclusive diagnosis after conventional genetic testing. By applying the computational method on larger cohorts of patients with de novo SVs, we estimated the contribution of position effects for both balanced and unbalanced SVs.

## Methods

### Patient selection and phenotyping

A total of 39 individuals with de novo germline SVs and an inconclusive diagnosis were included in this study. Individuals P1 to P21 and their biological parents were included at the University Medical Center Utrecht (the Netherlands) under study ID NL55260.041.15 15-736/M. Individual P22, previously described by Redin et al. as UTR22 [21], and her parents were included at the San Luigi University Hospital (Italy). For individuals P23 to P39, lymphoblastoid cell lines (LCL) were previously derived as part of the Developmental Genome Anatomy Project (DGAP) of the Brigham and Women’s Hospital and Massachusetts General Hospital, Boston, MA, USA [21]. Written informed consent was obtained for all included individuals and parents, and the studies were approved by the respective institutional review boards.

### DNA and RNA extraction

Peripheral blood mononuclear cells (PBMCs) were isolated from whole blood samples of individuals P1 to P22 and their biological parents using a Ficoll-Paque Plus gradient (GE Healthcare Life Sciences) in SepMate tubes (STEMCELL Technologies) according to the manufacturer’s protocols. LCL derived from individuals P23 to P39 were expanded in RPMI 1640 medium supplemented with GlutaMAX (Thermo Fisher Scientific), 10% fetal bovine serum, 1% penicillin, and 1% streptomycin at 37 °C. LCL cultures of each individual were split into three flasks and cultured separately for at least 1 week to obtain technical replicate samples for RNA isolation. Genomic DNA was isolated from the PBMCs or LCL using the QIASymphony DNA kit (Qiagen). Total RNA was isolated using the QIAsymphony RNA Kit (Qiagen), and RNA quality (RIN > 8) was determined using the Agilent RNA 6000 Nano Kit.

### Whole-genome sequencing

Purified DNA was sheared into fragments of 400–500 bp using a Covaris sonicator. WGS libraries were prepared using the TruSeq DNA Nano Library Prep Kit (Illumina). WGS libraries were sequenced on an Illumina Hiseq X instrument generating 2 × 150 bp paired-end reads to a mean coverage depth of at least × 30. The WGS data was processed using an in-house Illumina analysis pipeline (https://github.com/UMCUGenetics/IAP). Briefly, reads were mapped to the CRCh37/hg19 human reference genome using BWA-0.7.5a using “BWA-MEM -t 12 -c 100 -M -R” [22]. GATK IndelRealigner [23] was used to realign the reads. Duplicated reads were removed using Sambamba markdup [24].

### Structural variant calling and filtering

Raw SV candidates were called with Manta v0.29.5 using standard settings [25] and Delly v0.7.2 [26] using the following settings: “-q 1 -s 9 -m 13 -u 5.” Only Manta calls overlapping with breakpoint junctions called by Delly (± 100 bp) were selected. Rare SVs were selected by filtering against SV calls of 1000 Genomes [27] and against an in-house database containing raw Manta SV calls of ~ 120 samples (https://github.com/UMCUGenetics/vcf-explorer). De novo SVs were identified in individuals P1 to P22 by filtering the SVs of the children against the Manta calls (± 100 bp) of the father and the mother. Filtered SV calls were manually inspected in the Integrative Genome Viewer (IGV). The conformations of the complex derivative chromosomes were manually reconstructed based on genomic orientations of the filtered SV calls. De novo breakpoint junctions of individuals P1 to P21 were validated by PCR using AmpliTaq gold (Thermo Scientific) under standard cycling conditions and by Sanger sequencing. Primers were designed using Primer3 software (Additional file 1: Table S1). Breakpoint junction coordinates for individuals P22 to P39 were previously validated by PCR [21, 28].

### Single nucleotide variant filtering

Single nucleotide variants and indels were called using GATK HaplotypeCaller. For individuals P1 to P21 (whose parents were also sequenced), reads overlapping exons were selected and the Bench NGS Lab platform (Agilent-Cartagenia) was used to detect possible pathogenic de novo or recessive variants in the exome. The identified single nucleotide variants were classified according to the American College of Medical Genetics and Genomics (ACMG) criteria. De novo variants were only analyzed if they affect the protein structure of the genes that are intolerant to missense and loss-of-function variants. Only putative protein-changing homozygous and compound heterozygous variants with an allele frequency of < 0.5% in ExAC [29] were reported.

### RNA sequencing and analysis

RNA-seq libraries were prepared using TruSeq Stranded Total RNA Library Prep Kit (Illumina) according to the manufacturer’s protocol. RNA-seq libraries were pooled and sequenced on a NextSeq500 (Illumina) in 2 × 75 bp paired-end mode. Processing of RNA sequencing data was performed using a custom in-house pipeline (https://github.com/UMCUGenetics/RNASeq). Briefly, reads were aligned to the CRCh37/hg19 human reference genome using STAR 2.4.2a [30]. The number of reads mapping to genes were counted using HTSeq-count 0.6.1 [31]. Genes overlapping with SV breakpoints (e.g., truncated genes) were also analyzed separately by counting the number of reads mapping to exons per truncated gene fragment (up- and downstream of the breakpoint junction). RNA-seq data obtained from PBMCs (individuals P1 to P22) and LCL (individuals P23 to P39) were processed as separate datasets. The R-package DESeq2 was used to normalize raw read counts and to perform differential gene expression analysis for both datasets separately [32]. Genes with more than 0.5 reads per kilobase per million (RPKM) mapped reads were considered to be expressed.

### Gene annotation

Gene information (including genomic positions, Ensembl IDs, HGNC symbols, and Refseq IDs) was obtained from Ensembl (GRCh37) using the R-package biomaRt (v2.38) [33]. Genes containing a RefSeq mRNA ID and a HGNC symbol were considered as protein-coding genes. Genomic coordinates for the longest transcript were used if genes contained multiple RefSeq mRNA IDs. The list of 19,300 protein-coding genes was further annotated with (1) pLI, (2) RVIS, (3) haploinsufficiency (HI) and triplosensitivity scores, (4) OMIM identifiers, and (5) DDG2P information for each gene (see Additional file 1: Table S2 for data sources). These five categories were used to calculate a “disease association score” for each gene, which indicates if the gene has been associated with developmental disorders in general. Each gene was assigned one point per category if it met the following criteria (Table 1): (1) a pLI score of more than 0.9, (2) a RVIS score of less than 10, (3) a haploinsufficiency score of less than 10 or a ClinGen haploinsufficiency or triplosensitivity score between 1 and 3, (4) presence in the DDG2P database, and (5) presence in the OMIM database. Therefore, the disease association score ranges from 0 to 5, and a higher score indicates that the gene is associated with developmental disorders in multiple databases. Modes of inheritance for each gene (e.g., autosomal dominant, autosomal recessive, or X-linked) were retrieved from the HPO and DDG2P databases.

**Table 1:** Cutoffs used to classify affected genes as T1, T2, or T3 candidate driver genes

**1. Phenotype association**					
		*Weak*	*Medium*	*Strong*	
Disease association score (0–5)	pLI > 0.9 RVIS < 10 HI < 10 DDG2P OMIM	> 0	> 0	> 2	
Total phenomatch score		> 0	> 4	> 10	
Phenomatches (% of HPO terms with phenomatch score > 5)		> 0	> 10%	> 25%	
Mode of inheritance			AD/XD/XR+XY	AD/XD/XR+XY	
**2. Effect of SV on gene**					
		*Weak*		*Strong*	
Gene location		Adjacent	Dup	Adjacent	DEL/TRUNC
Support score (0–6)	TAD disrupted V4C disrupted PCHiC disrupted DHS disrupted RNA expression	> 1	NA	> 3	NA
**3. Driver classification**					
Classification		*T3*	*T2*		*T1*
Phenotype association + effect of SV on gene		Weak + weak	Strong + weak	Medium + strong	Strong + strong

*pLI* probability of being loss-of-function intolerant, *RVIS* Residual Variation Intolerance Score, *HI* haploinsufficiency, *DDG2P* Developmental Disorders Genotype-Phenotype Database, *OMIM* Online Mendelian Inheritance in Man, *AD* autosomal dominant, *XD* X-linked dominant, *XR* X-linked recessive, *XY* male, *TAD* topologically associating domain, *V4C* virtual 4C, *PCHiC* promoter capture Hi-C, *DHS* DNase hypersensitivity site

### Computational prediction of the effects of SVs on genes

For each patient, the protein-coding genes located at or adjacent (< 2 Mb) to the SVs were selected. The HPO terms linked to these genes in the HPO database were matched to each individual HPO term assigned to the patient and to the combination of the patient’s HPO terms. For each gene, the number of phenomatch scores higher than 1 (low phenomatches) and higher than 5 (high phenomatches) with individual patient HPO terms was calculated. The strength of the association (none, weak, medium, or strong) of each selected gene with the phenotype of the patient was determined based on the total phenomatch score, the number of low and high phenomatches, the mode of inheritance, and the disease association score (Table 1, Additional file 2: S1a).

Subsequently, potential direct and indirect effects of the SVs (none, weak, or strong) on the genes were predicted (Table 1, Additional file 2: Figure S1a). The prediction analyses were based on chromatin organization and epigenetic datasets of many different cell types obtained from previous studies (see Additional file 1: Table S2 for data sources).

First, we determined which TADs of 20 different cell types overlapped with the de novo SVs and which genes were located within these disrupted TADs [34–36] (Additional file 2: Figure S1b). To determine if the disrupted portions of the TADs contained regulatory elements that may be relevant for the genes located in the affected TADs, we selected the 3 cell types in which the gene is highly expressed based on RNA-seq data from the Encode/Roadmap projects [37] reanalyzed by Schmitt et al. [34] (Additional file 2: Figure S1C). The number of active enhancers (determined by chromHMM analysis of Encode/Roadmap ChIP-seq data [37]) in the TADs up- and downstream of the breakpoint junction in the 3 selected cell types was counted (Additional file 2: Figure SS1D). Virtual 4C was performed by selecting the rows of the normalized Hi-C matrices containing the transcription start site coordinates of the genes. The v4C profiles were overlapped with the breakpoint junctions to determine the portion of interrupted Hi-C interactions of the gene (Additional file 2: Figure S1e). In addition, promoter capture Hi-C data of 22 tissue types [38–41] and DNAse hypersensitivity site (DHS) connections [42] were overlapped with the SV breakpoints to predict disruption of long-range interactions over the breakpoint junctions (Additional file 2: Figure S1f). Genes with at least a weak phenotype association and a weak SV effect are considered as T3 candidate genes. Genes were classified as T1 candidate drivers if they have a strong association with the phenotype and are strongly affected by the SV. Genes classified as T2 candidate driver can have a weak/medium phenotype association combined with a strong SV effect or they can have a medium/strong phenotype association with a weak SV effect (Fig. 2a, Table 1).

### SV and phenotype information large patient cohorts

Breakpoint junction information and HPO terms for 228 individuals (excluding the individuals already included in this study for WGS and RNA-seq analysis) with mostly balanced SVs were obtained from Redin et al. [21]. Phenotype and genomic information for 154 patients with de novo copy number variants ascertained by clinical genomic arrays were obtained from an in-house patient database from the University Medical Center Utrecht (the Netherlands).

## Results

### WGS reveals hidden complexity of de novo SVs

**Fig. 1:**
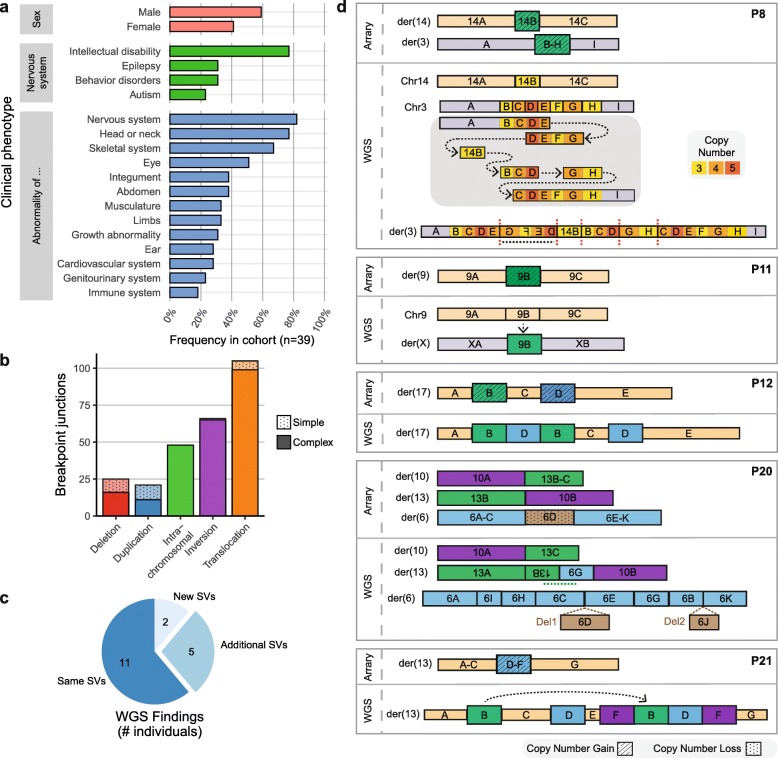
Characterization of de novo SVs in a cohort of individuals with neurodevelopmental disorders. **a** Frequencies of clinical phenotypic categories described for the 39 included individuals based on the categories defined by HPO. Nervous system abnormalities are divided into 4 subcategories. **b** Number of de novo breakpoint junctions per SV type identified by WGS of 39 included patients. Most detected de novo SVs are part of complex genomic rearrangements, which are defined by the involvement of more than 3 breakpoint junctions (SVs with 1 or 2 breakpoint junctions are considered simple rearrangements). **c** Number of cases in which WGS analysis identified new, additional, or similar SVs compared to microarray-based copy number profiling. **d** Schematic representation of additional genomic rearrangements that were observed by WGS in 5 individuals. For each patient, the top panel shows the de novo SVs identified by arrays or karyotyping and bottom panel shows the structures of the SVs detected by WGS. The WGS data of individual P8 revealed complex chromoanasynthesis rearrangements involving multiple duplications and an insertion of a fragment from chr14 into chr3. Individual P11 has an insertion of a fragment of chr9 into chrX that was detected as a copy number gain by array-based analysis (Additional file 2: Figure S2). The detected copy number gains in individuals P12 and P21 show an interspersed orientation instead of a tandem orientation. The translocation in patient P20 appeared to be more complex than previously anticipated based on karyotyping results, showing 11 breakpoint junctions on 3 chromosomes

We aimed to improve the genetic diagnosis of 39 individuals with multiple congenital abnormalities and/or intellectual disability (MCA/ID) who had an inconclusive diagnosis after regular genetic testing or who have complex genomic rearrangements. The phenotypes of the individuals were systematically described by Human Phenotype Ontology (HPO) terms [45–47]. The included individuals displayed a wide range of phenotypic features, and most individuals (82%) presented neurological abnormalities including intellectual disability (Fig. 1a, Additional file 1: Table S3) [21]. The parents of each of the patients were healthy, suggesting a de novo or recessive origin of the disease phenotypes. All individuals carried de novo SVs which were previously detected by ArrayCGH, SNP arrays, karyotyping, long-insert whole-genome sequencing, mate-pair sequencing, or targeted sequencing (Additional file 2: Figure S2a). First, we performed whole-genome sequencing (WGS) for all individuals in the cohort to screen for potential pathogenic genetic variants that were not detected by the previously performed genetic tests. No known pathogenic single nucleotide variants (SNVs) were detected in the individuals analyzed by patient-parents trio-based WGS (individuals P1 to P20), except for 1 pathogenic SNV that is associated with 1 component (hemophilia) of the comorbid phenotypic presentations of individual P1. A total of 46 unbalanced and 219 balanced de novo SVs were identified in the genomes of the individuals (Fig. 1b, Additional file 2: Figure S2b, Additional file 1: Table S1). The detected SVs ranged from simple SVs to very complex genomic rearrangements that ranged from 4 to 40 breakpoint junctions per individual. Importantly, WGS confirmed all previously detected de novo SVs and revealed additional complexity of the SVs in 7 (39%) of the 18 cases who were not studied by WGS-based techniques before (Fig. 1c, d; Additional file 1: Table S1). In half of the cases with previously identified de novo copy number gains (4/8), the gains were not arranged in a tandem orientation, but instead, they were inserted in another genomic region, which can have far-reaching consequences for accurate interpretation of the pathogenetic mechanisms in these individuals (Fig. 1d) [48–50]. This suggests that the complexity of copy number gains in particular is frequently underestimated by microarray analysis. For example, in one case (P11), a previously detected 170-kb copy number gain from chromosome 9 was actually inserted into chromosome X, 82 kb upstream of the *SOX3* gene (Fig. 1d, Additional file 2: Figure S3). This inserted fragment contains a super-enhancer region that is active in craniofacial development [51] (Additional file 2: Figure S3). The insertion of the super-enhancer may have disturbed the regulation of *SOX3* expression during palate development, which may represent a causal variant associated with the orofacial clefting in this individual [52–56]. The detection of these additional complexities in these seven patients exemplifies the added value that WGS analyses can have for cases that remain unresolved after standard array diagnostics [50].

### In silico phenomatching approach links directly affected genes to phenotypes

**Fig. 2:**
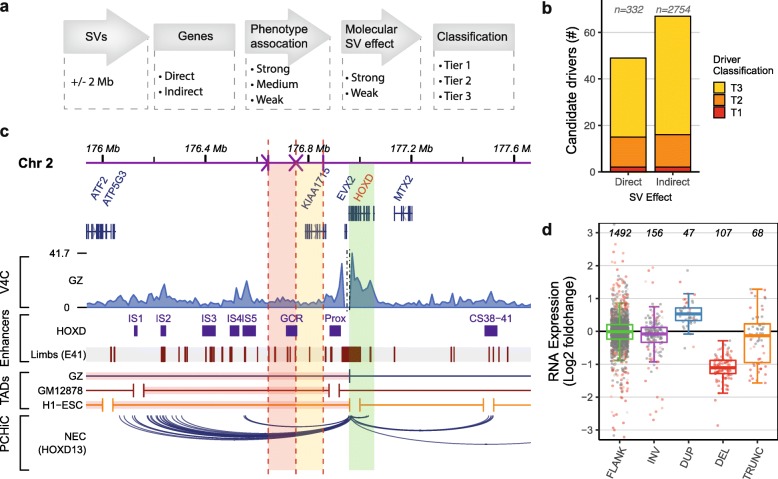
Prediction of candidate driver genes directly and indirectly affected by the SVs. **a** Schematic overview of the computational workflow developed to detect candidate driver genes. Classification of genes at (direct) or surrounding (indirect) the de novo SVs is based on the association of the gene with the phenotype and the predicted direct or indirect effect on the gene (Table 1). **b** Total number of identified tier 1, 2, and 3 candidate driver genes predicted to be directly or indirectly affected by an SV. **c** Genome browser overview showing the predicted disruption of regulatory landscape of the *HOXD* locus in individual P22. A 107-kb fragment (red shading) upstream of the *HOXD* locus (green shading) is translocated to a different chromosome, and a 106-kb fragment (yellow shading) is inverted. The SVs affect the TAD centromeric of the *HOXD* locus which is involved in the regulation of gene expression in developing digits. The translocated and inverted fragments contain multiple mouse [43] and human (day E41) [44] embryonic limb enhancers, including the global control region (GCR). Disruptions of these developmental enhancers likely contributed to the limb phenotype of the patient. The virtual V4C track shows the Hi-C interactions per 10 kb bin in germinal zone (GZ) cells using the *HOXD13* gene as viewpoint [35]. The bottom track displays the PCHiC interactions of the *HOXD13* gene in neuroectodermal cells [40]. UCSC Liftover was used to convert mm10 coordinates to hg19. **d** RNA expression levels of genes at or adjacent to de novo SVs. Log2 fold RNA expression changes compared to controls (see the “Methods” section) determined by RNA sequencing for expressed genes (RPKM > 0.5) that are located within 2 Mb of SV breakpoint junctions (FLANK) or that are inverted (INV), duplicated (DUP), deleted (DEL), or truncated (TRUNC). Differentially expressed genes (*p* < 0.05, calculated by DESeq2) are displayed in red

Subsequently, we determined if the phenotypes of the patients could be explained by direct effects of the de novo SVs, most of which were previously classified as a variant of unknown significance (VUS), on genes. In total, 332 genes are directly affected (deleted, duplicated, or truncated) by the de novo SVs in the cohort (Additional file 2: Figure S2c). The phenomatch tool was used to match the HPO terms associated with these genes with the HPO terms used to describe the phenotypes of the individuals [18, 19]. Genes were considered as candidate driver genes based on the height of their phenomatch score, the number of phenomatches between the HPO terms of the gene and the patient, recessive or dominant mode of inheritance, dosage sensitivity scores [57], loss-of-function constraint score (pLI) [29], Residual Variation Intolerance Score (RVIS) [58], and the presence in OMIM and/or DDG2P [59] databases (Table 1). Directly affected genes strongly or moderately associated with the phenotype are classified as tier 1 (T1) and tier 2 (T2) candidate driver genes, respectively (Fig. 2a, Table 1). Genes with limited evidence for contribution to the phenotype are reported as tier 3 (T3) genes. In the cohort of 39 patients, this approach prioritized 2 and 13 of the 332 directly affected genes as T1 and T2 candidate drivers, respectively (Fig. 2b). In 3 cases, the HPO terms of the identified T1/T2 candidate driver genes could be matched to more than 75% of the HPO terms assigned to the patients, indicating that the effects of the SVs on these genes can explain most of the phenotypes of these patients (Additional file 1: Table S4). In 6 other cases, directly affected T1/T2 candidate drivers were identified that were only associated with a part of the patient’s phenotypes (Additional file 1: Table S4).

Subsequently, we performed RNA sequencing on primary blood cells or lymphoblastoid cell lines derived from all 39 individuals to determine the impact of de novo SVs on RNA expression of candidate driver genes. RNA sequencing confirmed that most expressed genes directly affected by de novo deletions show a reduced RNA expression (97 of 107 genes with a median reduction of 0.46-fold compared to non-affected individuals) (Fig. 2d). Although duplicated genes show a median of 1.44-fold increase in expression, only 14 of 43 (~ 30%) of them are significantly overexpressed compared to the expression levels in non-affected individuals. In total, 87 genes are truncated by SVs and 4 of these are classified as T1/T2 candidate drivers. The genomic rearrangements lead to 12 possible fusions of truncated genes, and RNA-seq showed an increased expression for 2 gene fragments due to the formation of a fusion gene (Additional file 2: Figure S4, Additional file 1: Table S5). None of the genes involved in the formation of fusion genes were associated with the phenotypes of the patients, although we cannot exclude an unknown pathogenic effect of the newly identified fusion genes. We could detect expression for 3 deleted and 2 duplicated T1/T2 candidate drivers, and these were differentially expressed when compared to controls. The RNA sequencing data suggests that most genes affected by de novo deletions show reduced RNA expression levels and limited dosage compensation. However, increased gene dosage by de novo duplications does not always lead to increased RNA expression, at least in the blood cells of patients.

### Prediction of position effects of de novo SVs on neighboring genes

In 28 of the included cases (72%), our prioritization method did not predict T1/T2 candidate driver genes that are directly affected by the de novo SVs. Therefore, we investigated the position effects on the genes surrounding the de novo SVs to explain the phenotypes in those cases that were not fully explained by directly affected candidate driver genes. We extended our candidate driver gene prioritization analysis by including all the protein-coding genes located within 2 Mb of the breakpoint junctions, as most chromatin interactions are formed between the loci that are less than 2 Mb apart from each other [60]. Of the 2754 genes adjacent to the SVs, 117 are moderately to strongly associated with the specific phenotypes of the individuals based on the phenotype association analysis. However, this association with the phenotype does not necessarily mean that these genes located within 2 Mb of the breakpoint junctions are really affected by the SVs and thus contributing to the phenotype. To determine if the regulation of these genes was affected, we first evaluated the RNA expression levels of those genes. Three quarters (81/117) of the genes linked to the phenotypes were expressed, but only 9 of these showed reduced or increased expression (Fig. 2d). However, RNA expression in the blood may not always be a relevant proxy for most neurodevelopmental phenotypes [61, 62]. Therefore, we developed an extensive in silico strategy to predict potential disruption of the regulatory landscape of the genes surrounding the SVs (Additional file 2: Figure S1). Because the interactions between genes and their regulatory elements are cell type-specific, a large collection of tissue-specific Hi-C, TAD, promoter capture Hi-C (PCHiC), DNase hypersensitivity site (DHS), RNA, and ChIP-seq datasets was included (Additional file 1: Table S2). Several embryonic and neural cell type (such as fetal brain and neural progenitor cells) datasets were included that may be especially relevant to study the neurodevelopmental phenotypes in our cohort.

To predict potential disruption of the regulatory landscape of genes, we first selected for each of the assessed cell types the (1) TADs [34–36], (2) the PCHiC interactions [38–41], and (3) DHS connections [42] overlapping with the transcription start site of each gene adjacent to the SVs. We overlapped these gene-specific genome conformation features with the breakpoint junctions of the identified SVs to determine the proportion of disrupted genomic interactions for each gene (the “Methods” section, Additional file 2: Figure S1). We also counted the number of enhancers (which are active in cell types in which the genes show the highest RNA expression [37]) that are located on disrupted portions of the TADs. Additionally, we performed virtual 4C (v4C) for each gene by selecting the rows of the normalized Hi-C matrices containing the transcription start site coordinates of the genes as viewpoints, because the coordinates of TAD boundaries can be dependent on the calling method and the resolution of the Hi-C [63–65] and because a significant portion of genomic interactions crosses TAD boundaries [9]. Integrated scores for TAD disruption, v4C disruption, potential enhancer loss, disruption of PCHiC interactions, and DHS connections were used to calculate a position effect support score for each gene (Additional file 2: Figure S1). Finally, indirectly affected genes were classified as tier 1, 2, or 3 candidate drivers based on a combination of their association with the phenotype and their support score (Fig. 2a, Table 1).

Of the 117 genes that were associated with the phenotypes and located within 2 Mb of the SVs, 16 genes were predicted to be affected by the SVs based on the in silico analysis and therefore classified as T1/T2 candidate driver gene (Fig. 2b, Additional file 2: Figure S5). The validity of the approach was supported by the detection of pathogenic position effects identified in previous studies. For example, the regulatory landscape of *SOX9* was predicted to be disturbed by a translocation 721 kb upstream of the gene in individual P5, whose phenotype is mainly characterized by acampomelic campomelic dysplasia with Pierre-Robin syndrome (PRS) including a cleft palate (Additional file 2: Figure S6). SVs in this region have been predicted to disrupt interactions of *SOX9* with several of its enhancers further upstream, leading to phenotypes similar to the phenotype of individual P5 [66, 67]. In individual P39, who has been previously included in other studies, our method predicted a disruption of *FOXG1* expression regulation due to a translocation (Additional file 2: Figure S1), further supporting the hypothesis that deregulation of *FOXG1* caused the phenotype of this individual [21, 68].

Another example of a predicted position effect is the disruption of the regulatory landscape of the *HOXD* locus in individual P22. This individual has complex genomic rearrangements consisting of 40 breakpoint junctions on 4 different chromosomes likely caused by chromothripsis [28]. One of the inversions and 1 of the translocations are located in the TAD upstream (centromeric) of the *HOXD* gene cluster (Fig. 2c). This TAD contains multiple enhancers that regulate the precise expression patterns of the *HOXD* genes during the development of the digits [43, 69, 70]. Deletions of the gene cluster itself, but also deletions upstream of the cluster, are associated with hand malformations [71–73]. The translocation in individual P22 disrupts 1 of the main enhancer regions (the global control region (GCR)), which may have led to altered regulation of the expression of *HOXD* genes, ultimately causing brachydactyly and clinodactyly in this patient.

Our approach predicted position effects on T1/T2 candidate driver genes in ten included cases (26%) of which eight cases have balanced or complex SVs. This suggests that these effects may be especially important for balanced SVs.

### Prediction of driver genes improves molecular diagnosis

**Fig. 3:**
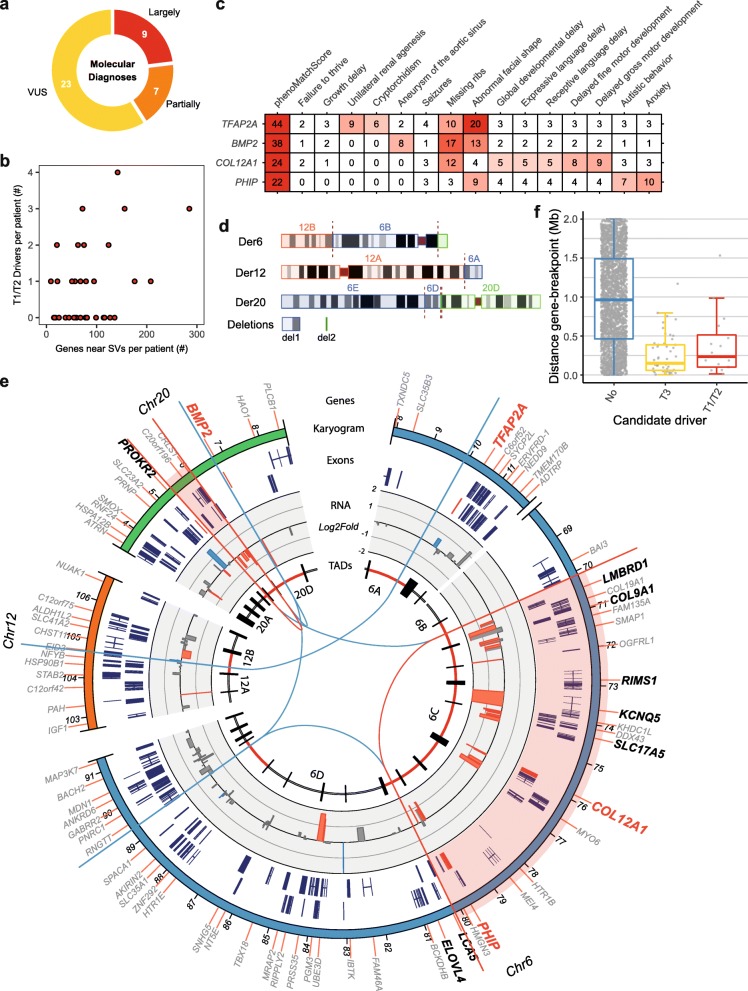
SVs can affect multiple candidate drivers which jointly contribute to a phenotype. **a** Number of patients whose phenotype can be partially or largely explained by the predicted T1/T2 candidate drivers (based on the percentage of the patient’s HPO terms that have a phenomatch score > 4). These molecular diagnoses are based on the fraction of HPO terms assigned to the patients that have a phenomatch score of more than 5 with at least one T1/T2 driver gene. **b** Scatterplot showing the number of predicted T1/T2 candidate drivers compared to the total number of genes at or adjacent (< 2 Mb) to the de novo SVs per patient. **c** Heatmap showing the association of the four predicted T1/T2 candidate drivers with the phenotypic features (described by HPO terms) of individual P25. The numbers correspond to the score determined by phenomatch. The four genes are associated with different parts of the complex phenotype of the patient. **d** Ideogram of the derivative (der) chromosomes 6, 12, and 20 in individual P25 reconstructed from the WGS data. WGS detected complex rearrangements with six breakpoint junctions and two deletions on chr6 and chr20 respectively of ~ 10 Mb and ~ 0.6 Mb. **e** Circos plot showing the genomic regions and candidate drivers affected by the complex rearrangements in individual P25. Gene symbols of T1/T2 and T3 candidate drivers are shown respectively in red and black. The breakpoint junctions are visualized by the lines in the inner region of the plot (red lines and highlights indicate the deletions). The middle ring shows the log2 fold change RNA expression changes in lymphoblastoid cells derived from the patient compared to controls measured by RNA sequencing. Genes differentially expressed (*p* < 0.05) are indicated by red (log2 fold change < − 0.5) and blue (log2 fold change > 0.5) bars. The inner ring shows the organization of the TADs and their boundaries (indicated by vertical black lines) in germinal zone (GZ) brain cells [35]. TADs overlapping with the de novo SVs are highlighted in red. **f** Genomic distance (in base pairs) between the indirectly affected candidate driver genes and the closest breakpoint junction. Most candidate drivers are located within 1 Mb of a breakpoint junction (median distance of 185 kb)

By combining both directly and indirectly affected candidate drivers per patient, we found possible explanations for the phenotypes of 16/39 (41%) complex and/or previously unsolved cases (Fig. 3a, Additional file 1: Table S4). Interestingly, in 8 cases, we found evidence for multiple candidate drivers that are individually only associated with part of the phenotype, but together may largely explain the phenotype (Fig. 3b). For example, we identified 4 candidate drivers in individual P25, who has a complex phenotype characterized by developmental delay, autism, seizures, renal agenesis, cryptorchidism, and an abnormal facial shape (Fig. 3c). This individual has complex genomic rearrangements consisting of 6 breakpoint junctions and 2 deletions of ~ 10 Mb and ~ 0.6 Mb on 3 different chromosomes (Fig. 3d). The 6q13q14.1 deletion of ~ 10 Mb affects 33 genes including the candidate drivers *PHIP* and *COL12A1*, which have been associated with developmental delay, anxiety, and facial dysmorphisms in other patients [74, 75]. In addition, 2 genes associated with other parts of the phenotype were predicted to be affected by position effects (Fig. 3e). One of these genes is *TFAP2A*, whose TAD (characterized by a large gene desert) and long-range interactions overlap with a translocation breakpoint junction. Rearrangements affecting the genomic interactions between *TFAP2A* and enhancers active in neural crest cells located in the *TFAP2A* TAD have recently been implicated in branchio-oculofacial syndrome [76]. The regulation of *BMP2*, a gene linked to agenesis of the ribs and cardiac features, is also predicted to be disturbed by a complex SV upstream of this gene [77, 78]. Altogether, these candidate driver genes may have jointly contributed to the phenotype of this individual (Fig. 3d). This case illustrates the challenge of identifying the causal genes driving the phenotypes of patients with structural rearrangements and highlights the notion that multiple genes should be considered for understanding the underlying molecular processes and explaining the patient’s phenotype [79].

### In silico driver gene prediction in larger patient cohorts

**Fig. 4:**
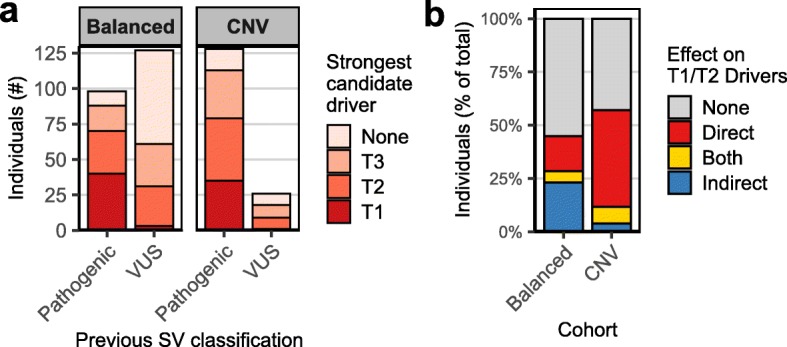
In silico prediction of candidate drivers in larger cohorts of patients with de novo SVs. **a** Comparison between previous SV classifications with the strongest candidate driver (located at or adjacent (< 1 Mb) to these SVs) predicted by our approach. Two different patient cohorts, one containing mostly balanced SVs [21] and one containing copy number variants, were screened for candidate drivers. Our method identified T1/T2 candidate drivers for most SVs previously classified as pathogenic or likely pathogenic. Additionally, the method detected T1/T2 candidate drivers for some SVs previously classified as VUS, which may lead to a new molecular diagnosis. **b** Quantification of the predicted effects of the SVs on proposed T1/T2 candidate driver genes per cohort. Individuals with multiple directly and indirectly affected candidate drivers are grouped in the category described as “Both.” Indirect position effects of SVs on genes contributing to phenotypes appear to be more common in patients with balanced SVs compared to patients with copy number variants

Our candidate driver prioritization approach identified many candidate drivers in previously unresolved cases, but these complex cases may not be fully representative for the general patient population seen in clinical genetic diagnostics. Therefore, we applied our prediction method to 2 larger sets of patients with de novo SVs to further assess the validity and value of the approach. We focused on the genes located at or within 1 Mb of the SVs, because most of the candidate driver genes we identified in our own patient cohort were located within 1 Mb of an SV breakpoint junction (Fig. 3f). First, we determined the effects of largely balanced structural variants in 225 previously described patients with varied congenital anomalies (Additional file 2: Figure S7a) [21]. In 98 of the 225 (44%) cases, the detected de novo SVs were previously classified as pathogenic or likely pathogenic, and in all but 3 of these diagnosed cases, 1 or more candidate driver genes have been proposed (Additional file 2: Figure S7b). Our approach identified 46 T1 and 97 T2 candidate drivers out of 7406 genes located within 1 Mb of the SVs (Additional file 2: Figure S7c,d; Additional file 1: Table S6). More than half (89/143) of the identified T1/T2 candidate drivers were not previously described as driver genes. In contrast, 22/114 (22%) previously described pathogenic or likely pathogenic drivers were classified as T3 candidates, and 38/114 (33%) were not reported as a driver by our approach (Fig. 4a), mostly because the phenomatch scores were below the threshold (46%) or because the genes were not associated with HPO terms (41%) (Additional file 2: Figure S7e). T1/T2 candidate drivers were identified in 101/225 (44%) of the individuals with mostly balanced SVs, including 31 individuals with SVs that were previously classified as VUS (Fig. 4b, Additional file 2: Figure S8). Position effect on genes moderately to strongly associated with the phenotypes was predicted in 64 (28%) of the cases with balanced SVs.

Subsequently, we also assessed the value of our driver prioritization approach for individuals with unbalanced copy number variants. We collected genetic and phenotypic information of 154 patients with a broad spectrum of (neuro-)developmental disorders who harbor de novo copy number variants (< 10 Mb) that were identified by clinical array-based copy number profiling (Additional file 2: Figure S7a,b; Additional file 1: Table S7). The CNVs in the majority (83%) of these individuals have been previously classified as pathogenic according to the clinical genetic diagnostic criteria (Additional file 2: Figure S7b). These criteria are mostly based on the overlap of the CNVs with CNVs of other individuals with similar phenotypes, and the causative driver genes were typically not previously specified. Our method identified T1/T2 candidate driver genes in 88/154 (57%) individuals, including 9/26 individuals with CNVs previously classified as VUS (Fig. 4a, Additional file 1: Table S6). Interestingly, support for position effects on candidate drivers was only found in 11% of the cases with CNVs, suggesting that pathogenic position effects are more common in patients with balanced SVs than in patients with unbalanced SVs (Fig. 4b). No driver genes were identified for 39% of the previously considered pathogenic CNVs (based on recurrence in other patients). In some cases, the potential drivers may remain unidentified because of incompleteness of the HPO database or insufficient description of the patient’s phenotypes. However, given the WGS results described for our patient cohort, it is also likely that some complexities of the CNVs may have been missed by the array-based detection method. The data also suggests that many disease-causing genes or mechanisms are still not known and that some SVs are incorrectly classified as pathogenic.

## Discussion

More than half of the patients with neurodevelopmental disorders do not receive a diagnosis after regular genetic testing based on whole-exome sequencing and microarray-based copy number profiling [3]. Furthermore, the molecular mechanisms underlying the disease phenotype often remain unknown, even when a genetic variant is diagnosed as (potentially) pathogenic in an individual, as this is often only based on recurrence in patients with a similar phenotype. Here, we applied an integrative method based on WGS, computational phenomatching and prediction of position effects to improve the diagnosis, and molecular understanding of the disease etiology of individuals with de novo SVs.

Our WGS approach identified additional complexities of the de novo SVs previously missed by array-based analysis in 7 of 18 cases, supporting previous findings that WGS can have an added value in identifying additional SVs that are not routinely detected by microarrays [50, 80, 81]. Our results indicate that duplications in particular are often more complex than interpreted by microarrays, which is in line with previous studies [48, 82]. WGS can therefore be a valuable follow-up method to improve the diagnosis particularly of patients with copy number gains classified as VUS. Knowing the exact genomic location and orientation of SVs is important for the identification of possible position effects.

To systematically dissect and understand the impact of de novo SVs, we developed a computational tool based on integration of HiC, RNA-seq, and ChIP-seq datasets to predict position effects of SVs on the regulation of gene expression. We combined these predictions with phenotype association information to identify candidate driver genes. In 9/39 of the complex cases, we identified candidate drivers that are directly affected by the breakpoint junctions of the SVs. Position effects of SVs have been shown to cause congenital disorders, but their significance is still unclear [14]. Our method predicted position effects on genes associated with the phenotype in 28% and 11% of all studied cases with balanced and unbalanced de novo SVs, respectively. Previous studies estimated that disruptions of TAD boundaries may be the underlying cause of the phenotypes of ~ 7.3% patients with balanced rearrangements [21] and of ~ 11.8% of patients with large rare deletions [18]. Our method identified a higher contribution of position effects in patients with balanced rearrangements mainly because our method included more extensive chromatin conformation datasets and also screened for effects that may explain smaller portions of the phenotypes. Our method, although it incorporates most of all published chromatin conformation datasets on untransformed human cells, focuses on the disruptions of interactions, which is a simplification of the complex nature of position effects. It gives an insight in the potential effects that lead to the phenotypes and prioritizes candidates that need to be followed up experimentally, ideally in a developmental context for proofing causality.

SVs can affect many genes, and multiple “disturbed” genes may together contribute to the phenotype. Indeed, in eight, cases we found support for the involvement of multiple candidate drivers that were affected by one or more de novo SVs. This supports previous findings that it can be important to consider multigenic effects to obtain a complete genetic diagnosis [79]. Such multigenic effects may be especially important for patients with large and complex SVs affecting many genes. This may underlie the relatively high amount of multigenic effects we predicted in our cohort compared to previous, mainly exome sequencing-based work that found a contribution of multilocus variation in 4.9% of cases [79]. In many of the studied cases, our method did not detect candidate drivers. This may be due to insufficient data or knowledge about the genes and regulatory elements in the affected locus and/or due to missing disease associations in the used databases. Additionally, de novo SVs are also frequently identified in healthy individuals in whom they do not have any pathogenic impact [83–85]. Some of the detected SVs of unknown significance may actually be benign and the disease caused by other genetic or non-genetic factors. The datasets underlying our computational workflow can be easily updated with more detailed data when emerging in the future, thereby enabling routine reanalysis of previously identified SVs. Moreover, our approach can be extended to study the consequences of SVs in different disease contexts such as cancer, where SVs also play a major causal role.

## Conclusions

Interpretation of SVs is important for clinical diagnosis of patients with developmental disorders, but it remains a challenge because SVs can have many different effects on multiple genes. We developed an approach to gain a detailed overview of the genes and regulatory elements affected by de novo SVs in patients with congenital disease. We show that WGS, if not available as a first-tier test, can be useful as a second-tier test to detect variants that are not detected by exome- and array-based approaches.

## Supplementary information

**Additional file 1:** **Table S1.** Coordinates of the de novo SV breakpoint junctions detected in the 39 individuals by WGS. **Table S2.** List of external data sources used in this study. **Table S3.** Phenotype information of the 39 included patients with de novo SVs. **Table S4.** Candidate driver genes detected for each included patient. **Table S5.** Fusion genes detected in the patients by RNA sequencing. **Table S6.** Candidate driver genes detected in patients’ cohorts. **Table S7**. Detected de novo copy number variants in 154 patients of the diagnostics cohort.

**Additional file 2.** Figure S1 to S8, including figure legends and supplemental references.

## Data Availability

Whole-genome sequencing and RNA sequencing datasets generated during the study have been deposited in the European Genome-phenome Archive under accession number EGAS00001003489 (https://www.ebi.ac.uk/ega/studies/EGAS00001003489) [86]. All custom code used in this study is available on https://github.com/UMCUGenetics/Complex_SVs [87].

## Abbreviations

HPO: Human Phenotype Ontology
RPKM: Reads per kilobase per million mapped reads
SNV: Single nucleotide variant
SV: Structural variant
TAD: Topologically associating domain
VUS: Variant of unknown significance
WGS: Whole-genome sequencing
