# Supplementary material for: Prioritization of genes driving congenital phenotypes of patients with de novo genomic structural variants
Source: Genome Med. 2019 Dec 4;11:79. doi: 10.1186/s13073-019-0692-0 (PMC6894143; doi:10.1186/s13073-019-0692-0)
Supplement: Supplementary file 2 — Additional file 2. Figure S1 to S8, including figure legends and supplemental references. [file 13073_2019_692_MOESM2_ESM.pdf]

Supplemental materials

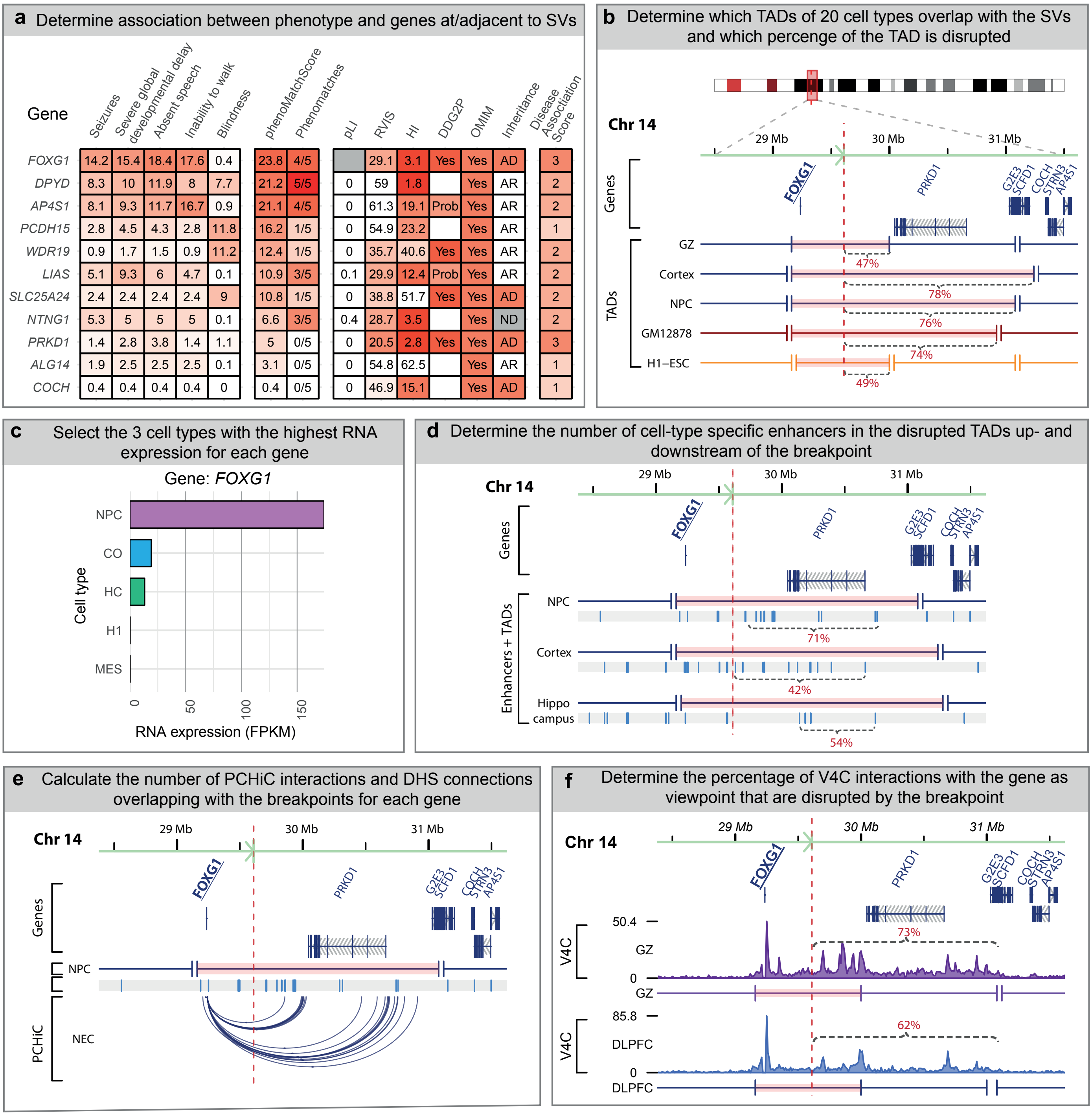

Fig. S1 Schematic overview of computational strategy used to predict position effects. **a** The association of a gene at or adjacent to an SV with the patient-specific phenotype is based on the phenomatch score, the number of phenomatches, mode of inheritance and the disease association score. Each gene has a fixed disease association score (ranging from 0 to 5) based on its pLI (>0.9), RVIS (<10) and haploinsufficiency (HI, <10) or triplosensitivity scores and the presence of the gene in DDG2P and OMIM. **b** The TADs of 20 different cell types are overlapped with the SV breakpoint junctions of the individual. The TADs affected by a breakpoint are split into fragments up- and downstream of the breakpoint and the size of each fragment (relative to the size of the intact TAD) is calculated. Subsequently the genes located on each fragment are determined and each gene receives a score based on the relative size of the fragment. For example, 47% of the TAD in germinal zone (GZ) cells containing *FOXG1* is considered disrupted. *(Continued on next page)*

c For each gene the 3 cell types with the highest RNA expression (FPKM: Fragments Per Kilobase Million) based on the Encode/Roadmap ChIP-seq data are selected. **d** For each gene, enhancers from the three selected cell types are overlapped with the disrupted TAD fragments. The number of enhancers in the disrupted part of the TAD is compared to the number of enhancers in the TAD fragment containing the gene. This ratio is considered at the percentage of enhancers moved away from the gene (for example, the location of 71% of neural progenitor cell enhancers in the *FOXG1* TAD is changed). **e** For each gene, PCHiC interactions of 22 cell types and promoter-DHS connections are overlapped with the breakpoint junctions. The number of interactions overlapping with the junctions is divided by the total number of interactions of the gene. For example, for *FOXG1* all 107 PCHiC interactions (in multiple cell types) overlap with the breakpoint junction. **f** Virtual 4C profiles were generated for each gene and these were overlapped with the breakpoint junctions to determine the percentage of interactions that are located up- or downstream of the breakpoint junction. For *FOXG1*, 73% of the V4C interactions in dorsolateral prefrontal cortex (DLPFC) cells are considered to be disrupted.

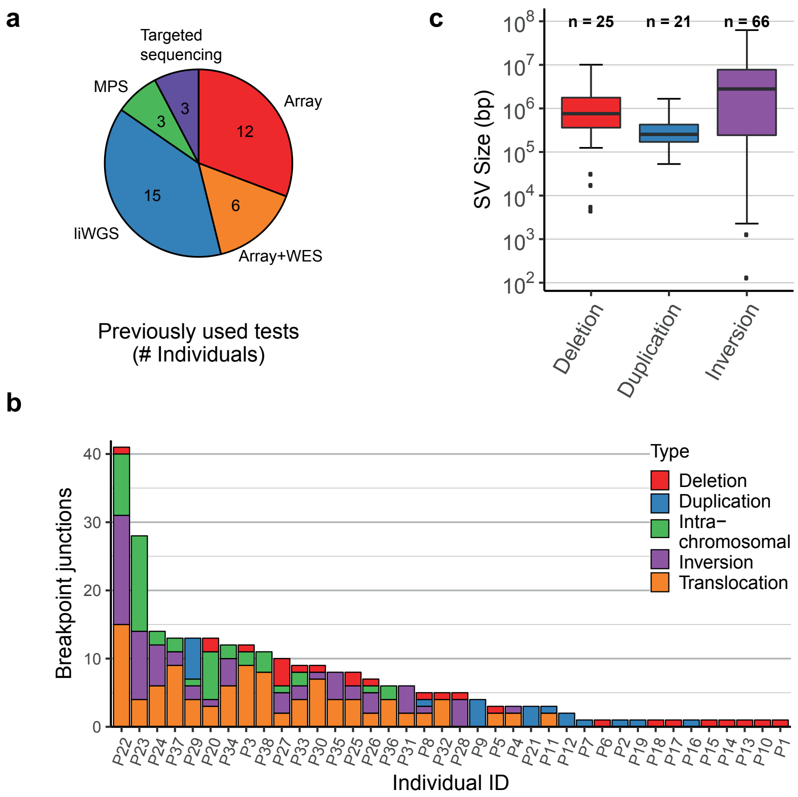

Fig. S2 Detected *de novo* germline SVs in 39 included patients. **a** Genetic tests previously used in a clinical setting to identify the *de novo* SVs in the included individuals. Microarrays (ArrayCGH or SNP arrays) were used to detect the deletions and duplications in 18 of the included individuals. MPS: Mate-pair sequencing, WES: Whole Exome Sequencing, liWGS: long-insert Whole Genome Sequencing. **b** Number of identified *de novo* SV breakpoint junctions per individual. **c** Size distribution in base pairs (bp) of the identified *de novo* deletions (median size 757,378 bp), duplications (median size 253,729 bp) and inversions (median size 2,295,988 bp).

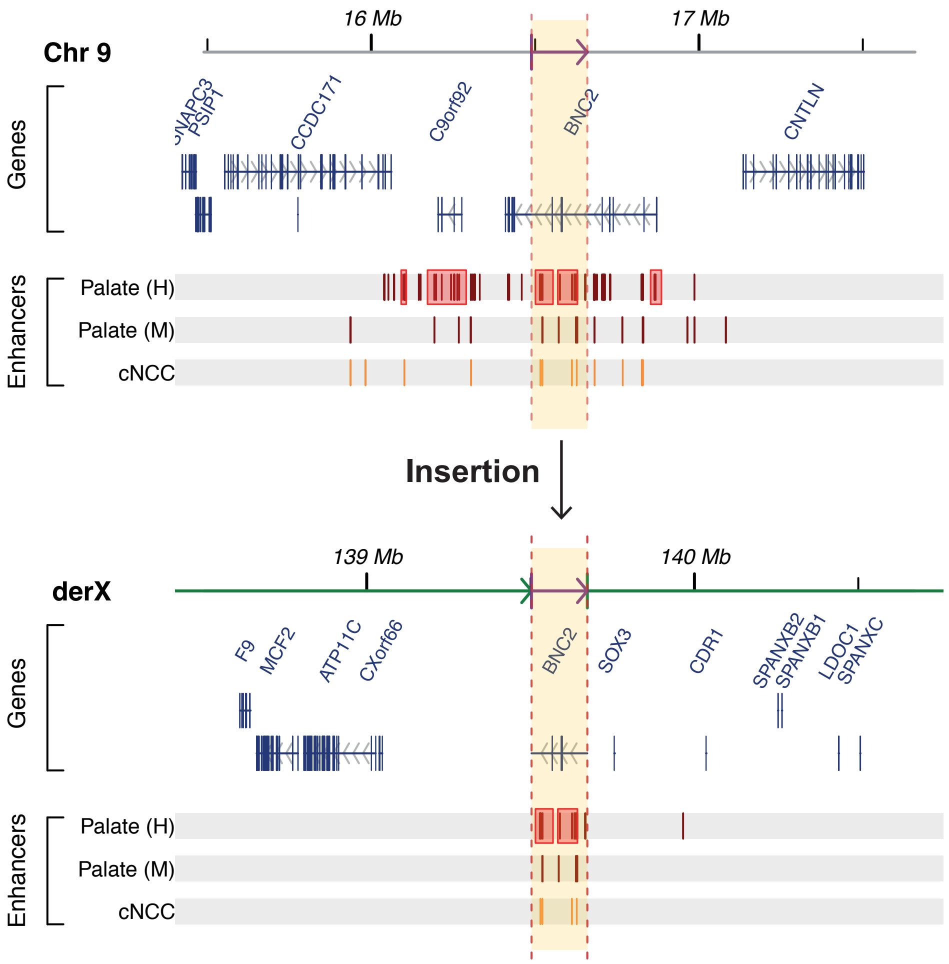

Fig. S3 Insertion of a super-enhancer region upstream of *SOX3* detected by WGS in individual P11. A 170kb duplication in the *BNC2* gene body at chr9 was reported by array-based analysis (top panel), but WGS detected that this duplication is actually inserted in chrX (bottom panel). The fragment (highlighted in yellow) is inserted 82 kb upstream of the *SOX3* gene. This locus at chrX contains a palindromic sequence that is susceptible for formation of genomic rearrangement. Multiple patients with varying phenotypes and different insertions at this locus have been described [1–5]. The inserted fragment from chr9 contains multiple enhancers, including two previously described super-enhancer clusters (highlighted by red boxes), that are active in human (Palate (H), Carnegie stage 13) and mouse (Palate (M), embryonic day 11.5) craniofacial development and human cultured cranial neural crest cells (cNCC) [6–8]. The inserted enhancers may disturb the normal expression of the *SOX3* gene and/or the surrounding genes, which may have led to the cleft palate phenotype in this patient. Genomic coordinates of mouse (mm9) embryonic craniofacial enhancers (determined by p300 ChIP-seq [6]) were converted to hg19 coordinates using LiftOver (https://genome.ucsc.edu/cgi-bin/hgLiftOver).

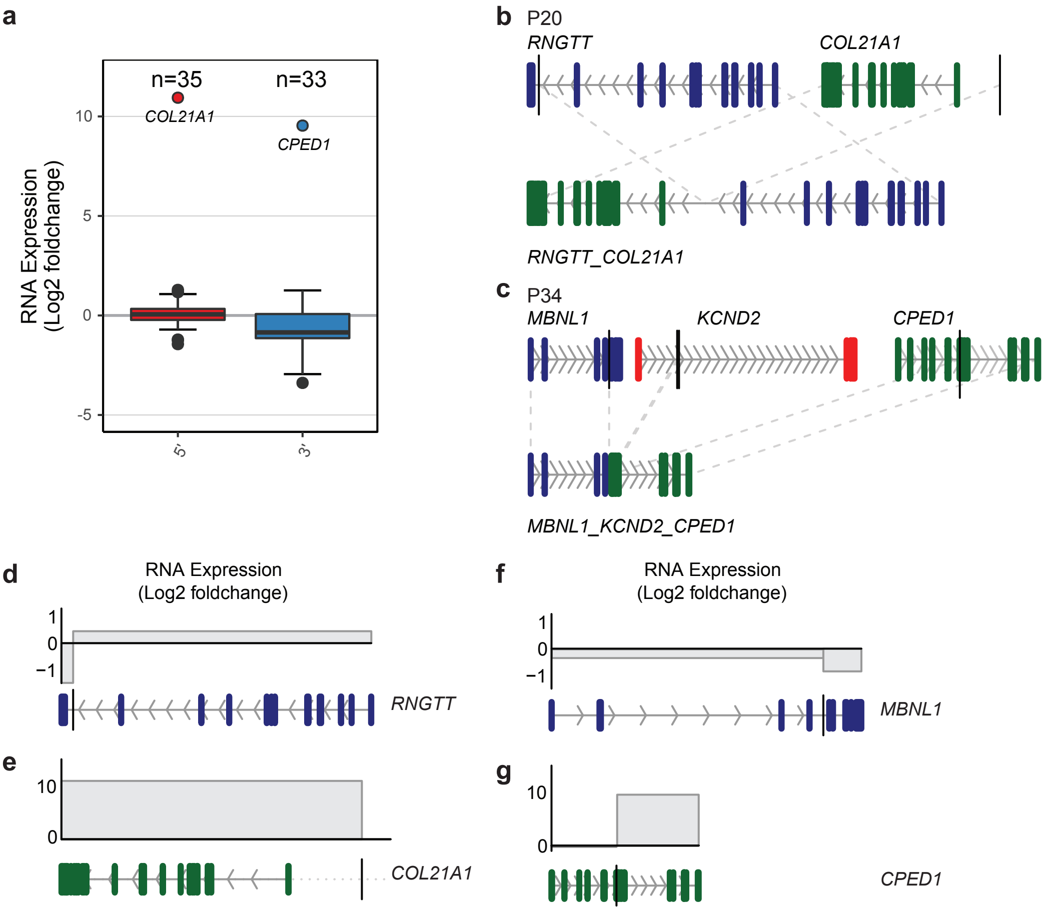

Fig. S4 RNA expression of genes truncated by de novo germline SVs. **a** Log2 fold change expression values (compared to expression of the exons in control individuals) for 5' gene fragments and 3' gene fragments of truncated genes. The 5' fragment of *COL21A1* and the 3' fragment of *CPED1* show a strong overexpression due to a gene fusion. **b** Schematic representation of the *RNGTT_COL21A1* fusion gene caused by genomic rearrangements in individual P20. The breakpoint junctions near the *RNGTT* (ENST00000369485) and *COL21A1* (ENST00000244728) gene bodies are depicted by the vertical black lines. **c** Schematic reconstruction of the *MBNL1_KCND2_CPED1* fusion gene in individual P34. Breakpoint junctions in the truncated genes *MBNL1* (ENST00000324210), *KCND2* (ENST00000331113) and *CPED1* (ENST00000310396) are represented by the vertical black lines. **d - g** RNA log2 fold change expression values (compared to the expression in unaffected individuals) for the fragments of the truncated genes *RNGTT*, *COL21A1*, *MLBNL1* and *CPED1*.

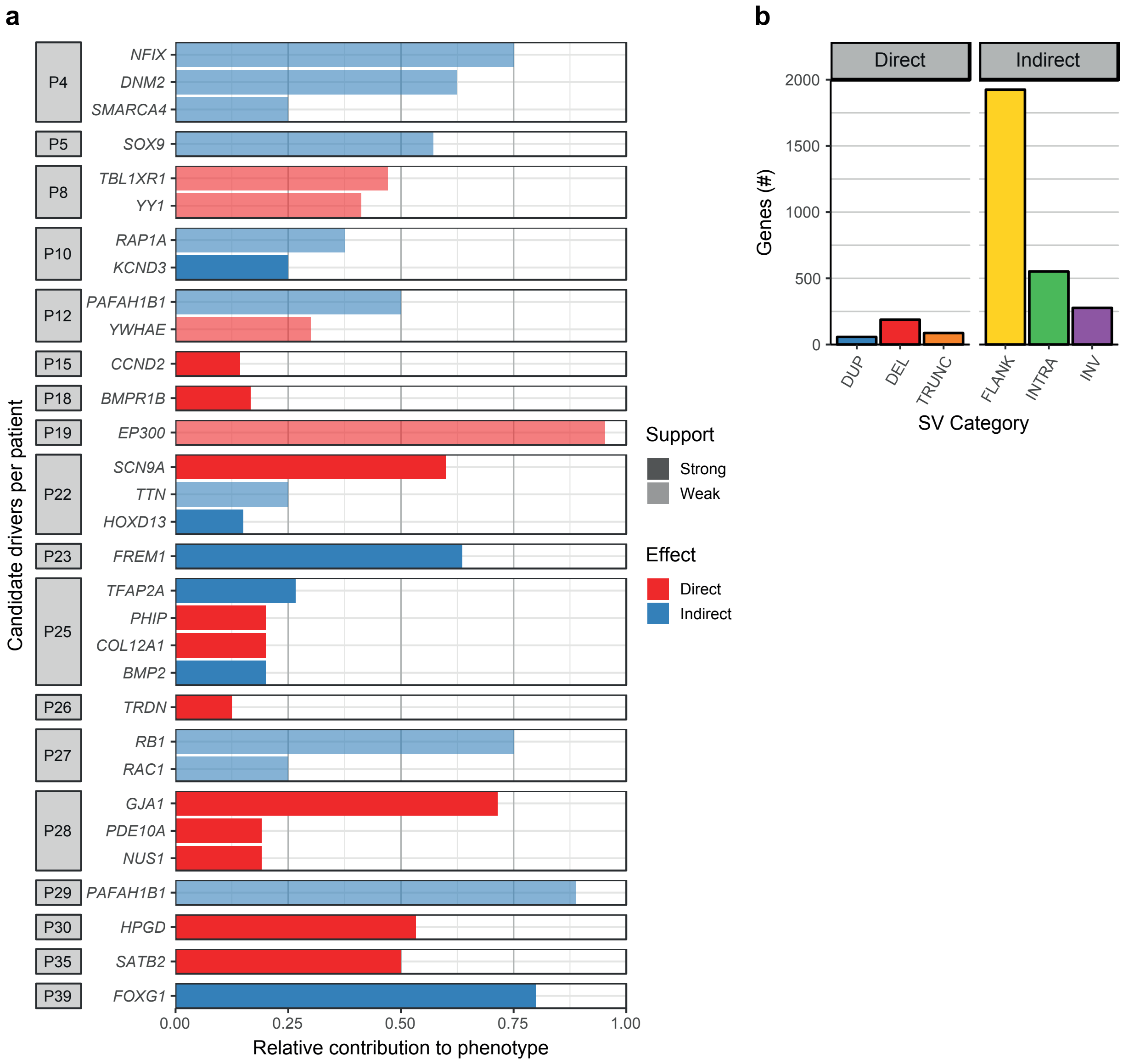

Fig. S5 Overview of the detected candidate driver genes. **a** Relative contributions of the candidate drivers to the phenotypes of the individuals. The contributions are based on the number of phenomatch hits (phenomatch score > 5) of a gene with each individual HPO term assigned to an individual, e.g. a gene with a contribution of 0.75 is associated with 75% of the HPO terms of an individual. Shading indicates if there is relatively weak or strong evidence for an effect on the candidate driver. **b** Total number of analysed genes per SV category. DUP: Duplication, DEL: Deletion, TRUNC: Truncation, FLANK: Flanking region (+/- 2Mb), Intra: Intrachromosomal rearrangement, INV: Inversion.

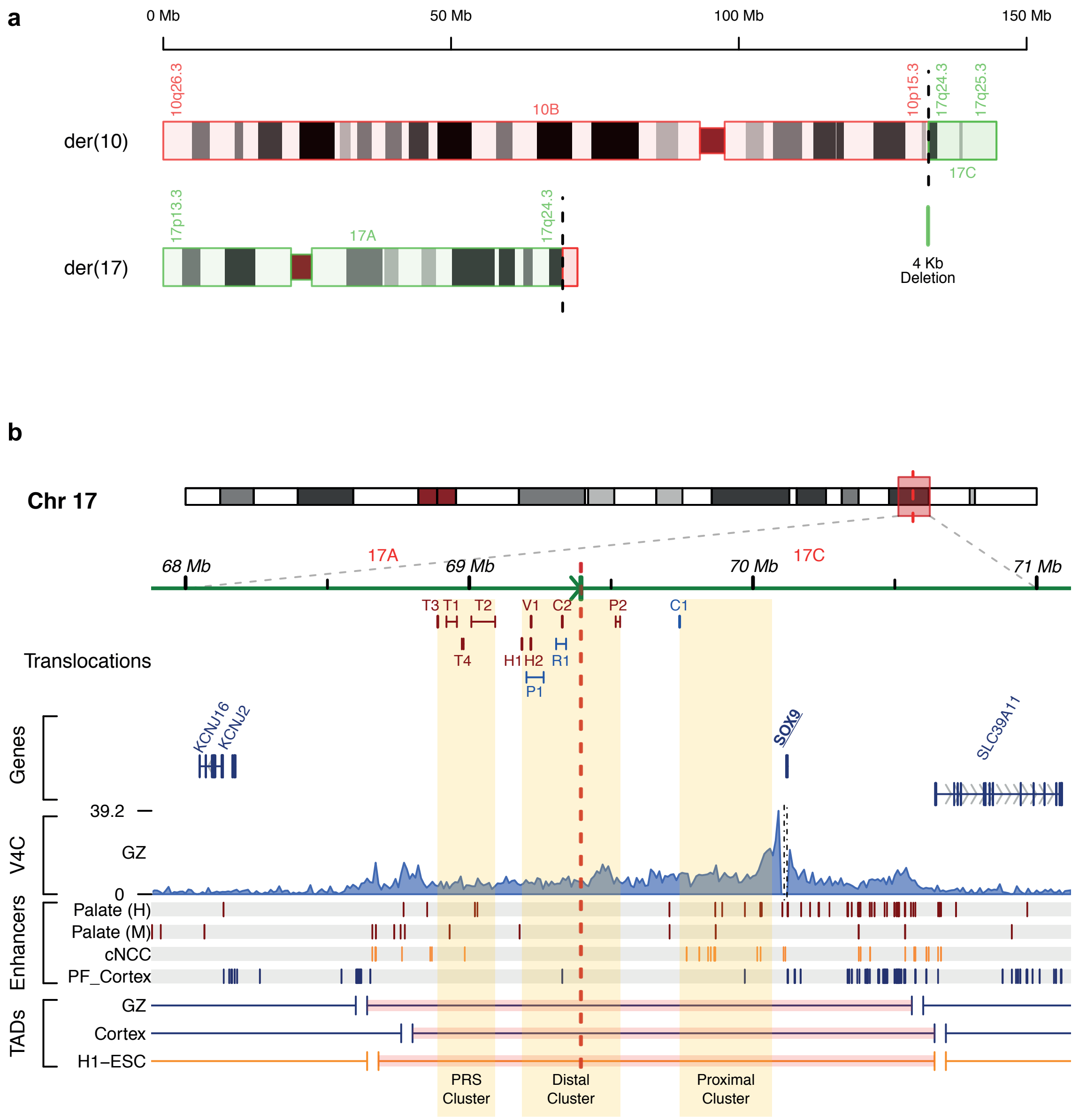

Fig. S6 Prediction of position effects of a translocation on SOX9 in individual P5. **a** Ideogram of the derivative chromosomes in individual P5. WGS identified a de novo translocation between chromosome 10 and 17 (46,XY,t(10;17)(p15;q24)). The breakpoint on chr17 (chr17:69395684, indicated by the vertical dotted red line) is 721 kb upstream of *SOX9*. A small 4kb fragment from chr17 is deleted (chr17:69391279-69395683). **b** Genome browser overview showing region surrounding the translocation breakpoint (red dotted line) at chromosome 17 in individual P5. The phenotype of this individual is characterized by acampomelic campomelic dysplasia and Pierre-Robin Syndrome including cleft palate, micrognathia and a long philtrum. SVs including translocations have been detected upstream of *SOX9* in individuals with various phenotypes including campomelic dysplasia. The translocations found in patients with phenotypes including cleft palate are shown in red and translocations found in patients with different phenotypes are depicted in blue. These translocations are predicted to separate *SOX9* from enhancers active in the developing palate, which may lead to the cleft palate phenotypes. Information about the other patients was obtained from the following publications: T1+T2+T3 [9]; T4 [10]; C1+C2 [11]; P1+P2 [12]; V1 [13]; R1 [14]; H1+H2 [15].

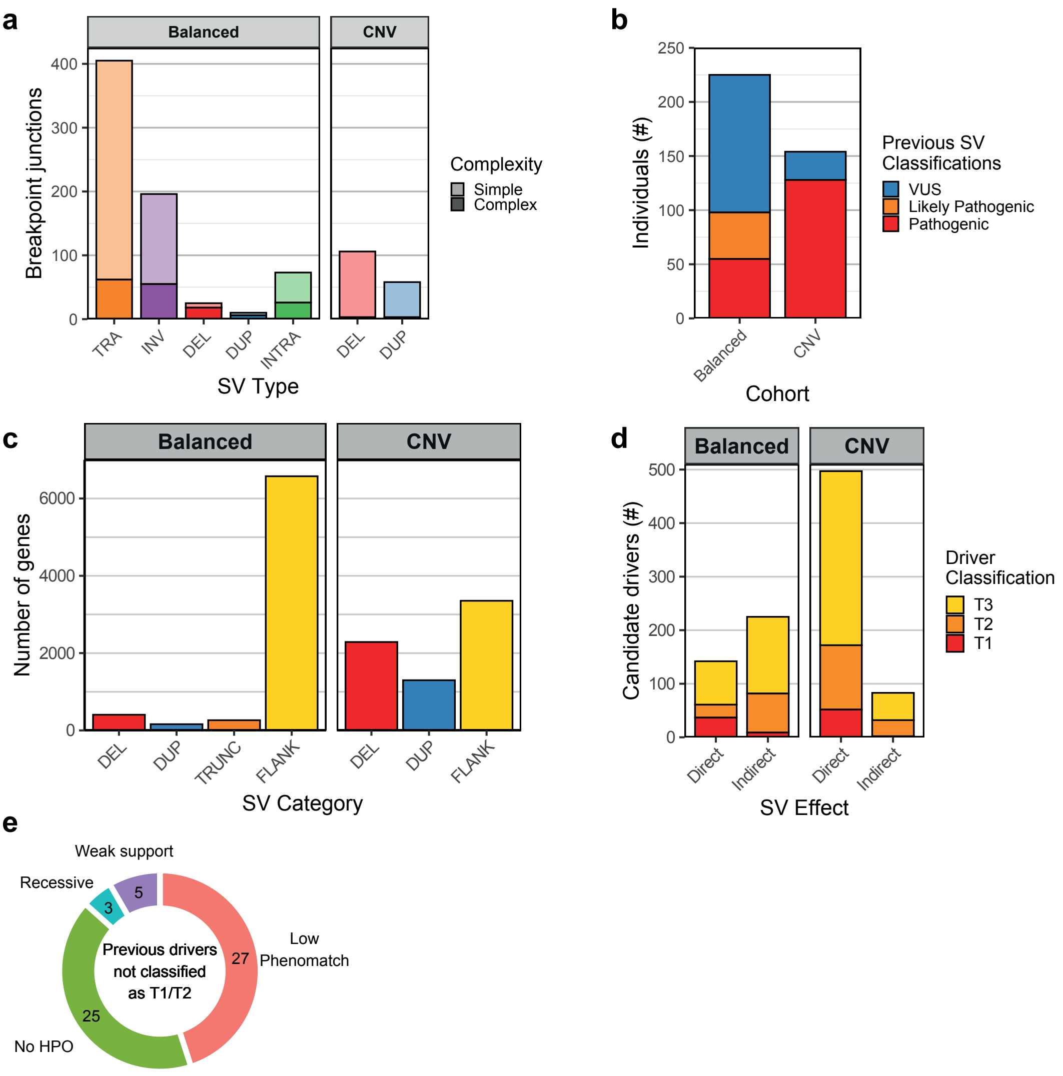

Fig. S7 Overview of SVs and candidate drivers in two cohorts of patients with *de novo* SVs. **a** Quantification of previously identified *de novo* SVs in a cohort containing patients with mostly balanced SVs and a cohort containing patients with copy number variants (CNV). *De novo* translocations (TRA), inversions (INV) and intra-chromosomal rearrangements (INTRA) are most prevalent in the cohort of patients with balanced SVs. Some patients have complex genomic rearrangements (>3 SVs) including some deletions (DEL) or duplications (DUP). The cohort labelled as "CNV" consist of patients with relatively simple deletions and duplications (<10 Mb in size). **b** Number of patients whose *de novo* SVs were previously classified as pathogenic, likely pathogenic or variant of unknown significance (VUS) per cohort. **c** Total number of analysed genes per SV category in the two cohorts. Dup: Duplicated, Del: Deleted, Trunc: Truncated, Flank: Flanking SVs (<1 Mb). **d** Total number of predicted directly and indirectly affected candidate drivers per cohort. **e** Quantification of the genes that were previously classified as pathogenic or likely pathogenic (by Redin et al [16]), but not identified as T1 or T2 candidate driver by our approach. These classification differences may be caused by a lack of HPO terms associated with the gene, low phenomatch scores below the threshold of our method, insufficient (weak) support for an effect of an SV on the gene detected by our method or a presumed recessive mode of inheritance.

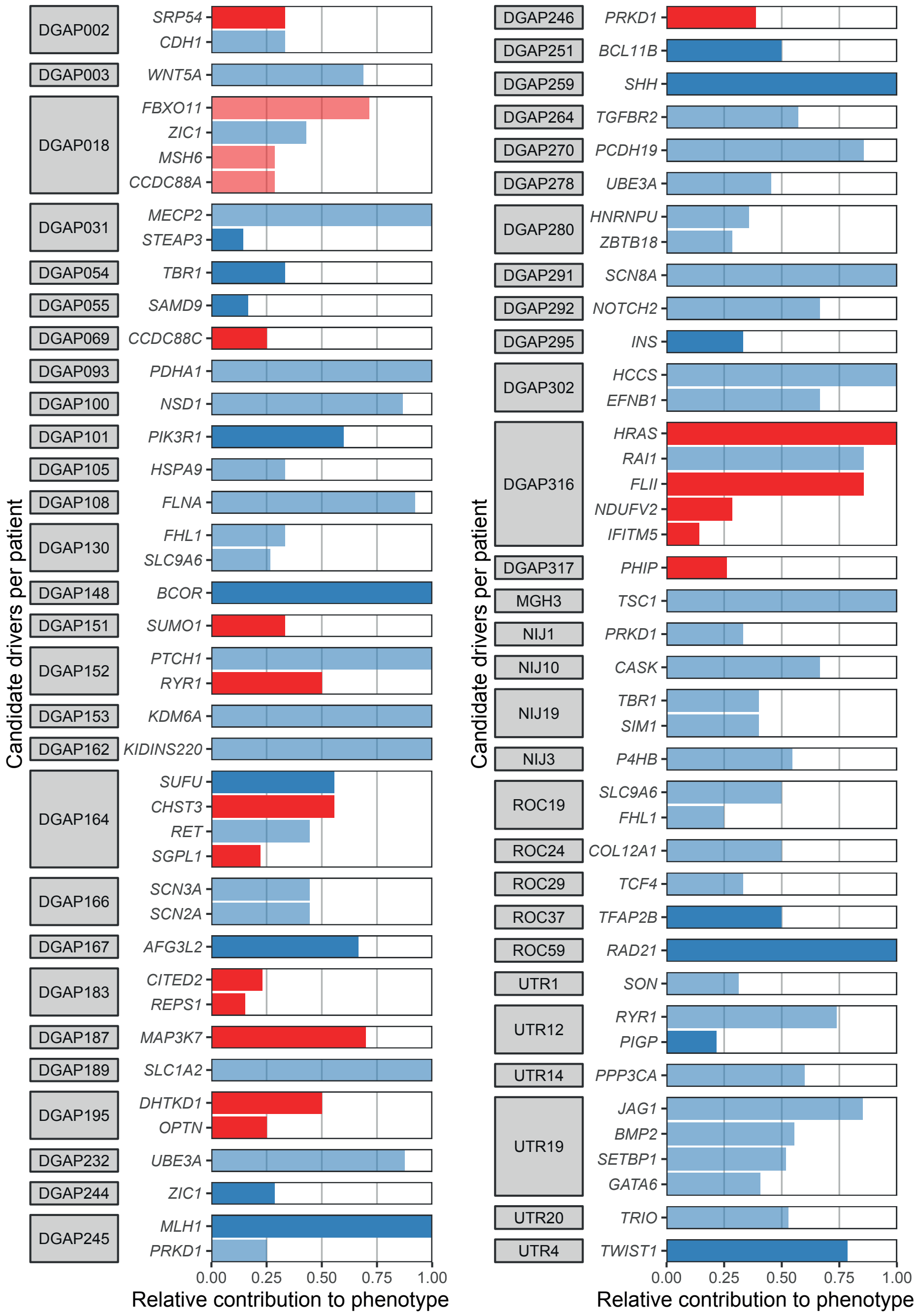

Fig. S8 Predicted contributions of candidate drivers to the phenotypes of patients with balanced structural variants of unknown significance. T1/T2 candidate drivers were detected in 31 patients whose *de novo* SVs were previously classified as VUS by Redin et al [16]. The contributions to the phenotypes are based on the number of phenomatch hits (phenomatch score > 5) of the gene with each individual HPO term used to describe the phenotype of a patient. Shading indicates if there is relatively weak or strong evidence for an effect on the candidate driver.

Supplemental references

1. Brewer MH, Chaudhry R, Qi J, Kidambi A, Drew AP, Menezes MP, et al. Whole Genome Sequencing Identifies a 78 kb Insertion from Chromosome 8 as the Cause of Charcot-Marie-Tooth Neuropathy CMTX3. PLoS Genet. 2016;12:e1006177.

2. Haines B, Hughes J, Corbett M, Shaw M, Innes J, Patel L, et al. Interchromosomal insertional translocation at Xq26.3 alters SOX3 expression in an individual with XX male sex reversal. J Clin Endocrinol Metab. 2015;100:E815–20.

3. Bunyan DJ, Robinson DO, Tyers AG, Huang S, Maloney VK, Grand FH, et al. X-Linked Dominant Congenital Ptosis Cosegregating with an Interstitial Insertion of a Chromosome 1p21.3 Fragment into a Quasipalindromic Sequence in Xq27.1. OJGen. 2014;04:415–25.

4. DeStefano GM, Fantauzzo KA, Petukhova L, Kurban M, Tadin-Strapps M, Levy B, et al. Position effect on FGF13 associated with X-linked congenital generalized hypertrichosis. Proc Natl Acad Sci U S A. 2013;110:7790–5.

5. Zhu H, Shang D, Sun M, Choi S, Liu Q, Hao J, et al. X-linked congenital hypertrichosis syndrome is associated with interchromosomal insertions mediated by a human-specific palindrome near SOX3. Am J Hum Genet. 2011;88:819–26.

6. Attanasio C, Nord AS, Zhu Y, Blow MJ, Li Z, Liberton DK, et al. Fine tuning of craniofacial morphology by distant-acting enhancers. Science. 2013;342:1241006.

7. Prescott SL, Srinivasan R, Marchetto MC, Grishina I, Narvaiza I, Selleri L, et al. Enhancer divergence and cis-regulatory evolution in the human and chimp neural crest. Cell. 2015;163:68–83.

8. Wilderman A, VanOudenhove J, Kron J, Noonan JP, Cotney J. High-Resolution Epigenomic Atlas of Human Embryonic Craniofacial Development. Cell Rep. 2018;23:1581–97.

9. Benko S, Fantes JA, Amiel J, Kleinjan D-J, Thomas S, Ramsay J, et al. Highly conserved non-coding elements on either side of SOX9 associated with Pierre Robin sequence. Nat Genet. 2009;41:359–64.

10. Jakobsen LP, Ullmann R, Christensen SB, Jensen KE, Mølsted K, Henriksen KF, et al. Pierre Robin sequence may be caused by dysregulation of SOX9 and KCNJ2. J Med Genet. 2007;44:381–6.

11. Leipoldt M, Erdel M, Bien-Willner GA, Smyk M, Theurl M, Yatsenko SA, et al. Two novel translocation breakpoints upstream of SOX9 define borders of the proximal and distal breakpoint cluster region in campomelic dysplasia. Clin Genet. 2007;71:67–75.

12. Fonseca ACS, Bonaldi A, Bertola DR, Kim CA, Otto PA, Vianna-Morgante AM. The clinical impact of chromosomal rearrangements with breakpoints upstream of the SOX9gene: two novel de novo balanced translocations associated with acampomelic campomelic dysplasia. BMC Med Genet [Internet]. 2013;14. Available from: http://dx.doi.org/10.1186/1471-2350-14-50

13. Velagaleti GVN, Bien-Willner GA, Northup JK, Lockhart LH, Hawkins JC, Jalal SM, et al. Position effects due to chromosome breakpoints that map approximately 900 Kb upstream and approximately 1.3 Mb downstream of SOX9 in two patients with campomelic dysplasia. Am J Hum Genet. 2005;76:652–62.

14. Refai O, Friedman A, Terry L, Jewett T, Pearlman A, Perle MA, et al. De novo 12;17 translocation upstream of SOX9 resulting in 46,XX testicular disorder of sex development. Am J Med Genet A. 2010;152A:422–6.

15. Hill-Harfe KL, Kaplan L, Stalker HJ, Zori RT, Pop R, Scherer G, et al. Fine mapping of chromosome 17 translocation breakpoints > or = 900 Kb upstream of SOX9 in acampomelic campomelic dysplasia and a mild, familial skeletal dysplasia. Am J Hum Genet. 2005;76:663–71.

16. Redin C, Brand H, Collins RL, Kammin T, Mitchell E, Hodge JC, et al. The genomic landscape of balanced cytogenetic abnormalities associated with human congenital anomalies. Nat Genet. 2017;49:36–45.

17. Cotney J, Leng J, Yin J, Reilly SK, DeMare LE, Emera D, et al. The evolution of lineage-specific regulatory activities in the human embryonic limb. Cell. 2013;154:185–96.

18. Monti R, Barozzi I, Osterwalder M, Lee E, Kato M, Garvin TH, et al. Limb-Enhancer Genie: An accessible resource of accurate enhancer predictions in the developing limb. PLoS Comput Biol. 2017;13:e1005720.
